# Stemming the Tide of Antibiotic Resistance (STAR): A protocol for a trial of a complex intervention addressing the 'why' and 'how' of appropriate antibiotic prescribing in general practice

**DOI:** 10.1186/1471-2296-10-20

**Published:** 2009-03-23

**Authors:** Sharon A Simpson, Christopher C Butler, Kerry Hood, David Cohen, Frank Dunstan, Meirion R Evans, Stephen Rollnick, Laurence Moore, Monika Hare, Marie-Jet Bekkers, John Evans, The STAR study team

**Affiliations:** 1 South East Wales Trials Unit, School of Medicine, Cardiff University, 7th floor Neuadd Meirionnydd, Heath Park, Cardiff, CF14 4XN, UK; 2 Department of Primary Care and Public Health, School of Medicine, Cardiff University, 3rd floor Neuadd Meirionnydd, Heath Park, Cardiff, CF14 4XN, UK; 3 Health Economics and Policy Research Unit, University of Glamorgan, Pontypridd, CF37 1DL, UK; 4 Cardiff Institute of Society, Health and Ethics, School of Social Science, Cardiff University, 53 Park Place, Cardiff, CF10 3AT, UK

## Abstract

**Background:**

After some years of a downward trend, antibiotic prescribing rates in the community have tended to level out in many countries. There is also wide variation in antibiotic prescribing between general practices, and between countries. There are still considerable further gains that could be made in reducing inappropriate antibiotic prescribing, but complex interventions are required. Studies to date have generally evaluated the effect of interventions on antibiotic prescribing in a single consultation and pragmatic evaluations that assess maintenance of new skills are rare. This paper describes the protocol for a pragmatic, randomized evaluation of a complex intervention aimed at reducing antibiotic prescribing by primary care clinicians.

**Methods and design:**

We developed a Social Learning Theory based, blended learning program (on-line learning, a practice based seminar, and context bound learning) called the STAR Educational Program. The 'why of change' is addressed by providing clinicians in general practice with information on antibiotic resistance in urine samples submitted by their practice and their antibiotic prescribing data, and facilitating a practice-based seminar on the implications of this data. The 'how of change' is addressed through context-bound communication skills training and information on antibiotic indication and choice. This intervention will be evaluated in a trial involving 60 general practices, with general practice as the unit of randomization (clinicians from each practice to either receive the STAR Educational Program or not) and analysis. The primary outcome will be the number of antibiotic items dispensed over one year. An economic and process evaluation will also be conducted.

**Discussion:**

This trial will be the first to evaluate the effectiveness of this type of theory-based, blended learning intervention aimed at reducing antibiotic prescribing by primary care clinicians. Novel aspects include feedback of practice level data on antimicrobial resistance and prescribing, use of principles from motivational interviewing, training in enhanced communication skills that incorporates context-bound experience and reflection, and using antibiotic dispensing over one year (as opposed to antibiotic prescribing in a single consultation) as the main outcome.

**Trial registration:**

Current Controlled Trials ISRCTN63355948.

## Background

Antibiotic resistance is one of the most important public health issues of our time[1,2]. The reservoir of microbial sensitivity is a precious international resource that is being depleted[3]. No completely novel class of antibiotics are expected on the market for primary care in the medium term, and the pipeline for new gram negative chemotherapy is particularly bleak[4]. Therefore, conserving antibiotic sensitivity through more appropriate antibiotic use is a priority[5]. In addition to driving resistance, inappropriate prescribing wastes money, exposes people to unnecessary side effects, and encourages future consulting[6,7]. We have shown that antibiotic resistance is common in primary care and causes symptoms to last for longer in primary care patients[8] and that patients with resistant infections incur higher drug and reconsultation costs[9]. Reduced antibiotic prescribing has been shown to be associated with reduced levels of resistance both at a national and local level [10-12].

About 85–90% of all antibiotics are prescribed in primary care and about 50% of prescription are of questionable value[13]. In the UK, after some years of a downward trend in the number of antibiotics dispensed to ambulatory patients, rates have levelled out[3]. There is wide variation both within and between countries in antibiotic prescribing rates that cannot be explained by differences in the epidemiology of infections[3,14,15]. The UK prescribed nearly twice the amount of antibiotics to ambulatory patients as the Netherlands[3]. This suggests that considerable further gains could safely be made in reducing inappropriate antibiotic prescribing both at practice level and nationally.

Whilst previous interventions aimed at general practitioners have been successful in reducing antibiotic prescribing with single consultations as the unit of study[16,17], these studies have generally achieved small effect sizes, partly because of low intensity, inflexible interventions that have often been focused only on generalities about why change is important[16]. Studies that randomize individual patients assess prescribing outcomes in single consultations, and so do not assess maintenance of new skills. Behaviour in a consultation that is sharply under a research spotlight may be quite different to prescribing behaviour over a whole year. This study builds on a considerable amount of development work and integrates practice antibiotic prescribing and antimicrobial resistance data with innovative developments in the fields of microbiology, prescribing, education, communication and behavioural sciences, into a single, yet flexible intervention. The effects will be measured using routinely collected data over a whole year.

We have developed an educational program that integrates a practice-based seminar with on-line learning, followed by a maintenance phase. The STAR program aims to enhance communication within the consultation and to promote behaviour change by both patients (more appropriate self-care, fewer antibiotics) and clinicians (enhanced communication skills, reduced prescribing). Behaviour change theory is important to both outcomes.

### Behaviour change theory

Social Learning Theory makes the critical distinction between outcome and efficacy expectations, and the evidence in support of verbal persuasion, modelling and mastery experiences for enhancing self-efficacy[18,19]. Similarly, research on the Theory of Planned Behaviour[20,21] makes a conceptual distinction between beliefs about consequences and beliefs about the ability to exert control over change. A systematic review of behaviour change interventions based on the Theory of Planned Behaviour found that two thirds of such interventions were effective[21] and a meta-analysis provides supporting evidence[22]. Taken together, these theories and associated research provide the foundation for the key elements of the STAR Program: behaviour change will be more likely if an intervention addresses both the *'why' *(importance of change; outcome expectations; beliefs about consequences) and the *'how' *(confidence in making changes; self-efficacy; beliefs about control).

### Other evidence

The large body of research on clinician behaviour change is congruent with this evidence from psychological theory. Put simply, verbal persuasion alone does not usually alter beliefs about the importance of change or enhance mastery over efforts to change the consultation itself. Practice guidelines also have a limited effect on practitioner behaviour change even when the clinicians have been involved in their development [23,24]. Over 20 systematic reviews [25-27] and reviews of them[28,29] suggest that behaviour change interventions should be multifaceted, assess barriers to change, be responsive to local circumstances, have a focused and active educational outreach component, including skills development, and be resonant with clinicians' values[30,31].

A systematic review identified four randomized controlled trials comparing a delayed script to an immediate script for respiratory tract infections and found a relative risk for lower antibiotic use when a delayed script was issued of 0.54 for the common cold and 0.25 for otitis media[32]. A systematic review (completed as part of a PhD thesis) of interventions aimed at optimizing antibiotic prescribing for acute respiratory tract infections in primary care identified 8 studies evaluating 12 interventions[16]. Studies were of low quality with modest effect sizes (mean of 6% reduction in prescription rates). Welschen's own subsequent study[33] achieved a much greater effect size of 12% reduction, with involvement of GPs at a local level. However, this study differs in a number of crucial respects to our proposed study. The intervention did not identify specific strategies, or demonstrate communication skills, or provide actual training in skill acquisition. The face-to-face meetings simply mentioned communication, with no subsequent requirement to practice and reflect on the use of specific strategies.

The STAR Educational Program has been developed in consultation with clinicians in order to be multi-faceted (e-learning, outreach, experiential), responsive to local circumstances (feedback of practice level data on prescribing and resistance), based on our previous work on everyday frustrations, challenges and barriers to change (practice-wide effort; time-efficient consultation strategies that 'enable' patients and maintain relationships) [34,35] and skills-based. It is also congruent with the guidance of Grol and colleagues[36] in its use of a phased engagement and implementation process to achieve certification and then to maintain change through web-based exchange of experiences.

### The 'why' of change

The 'why' of clinician behaviour change is addressed not only by exposure to evidence and opinions, but by encouraging clinicians to make sense of their prescribing and microbiological data at a locally meaningful, practice level. Some clinicians in qualitative studies have suggested that their prescribing behaviour has no impact on resistance [37-39]. Wales has a novel system that links data on dispensed antibiotics with antimicrobial sensitivity test results [40]. The STAR program will feed back this information at a practice level as a means of raising discussion and enhancing motivation to change prescribing[27].

### The 'how' of change

Clinicians report that they find it hard not prescribing an antibiotic while maintaining good relationships with patients in time-pressured consultations[41]. While newer work suggests that clinicians find antibiotic prescribing 'less uncomfortable'[42], it is certain that interventions are more likely to be taken up if they are time efficient and acceptable to both clinicians and patients[42]. Clinicians have confirmed that they need feasible and more effective communication strategies to successfully change their prescribing[38]. Patients with common infections consult with a variety of expectations and may go away with these unfulfilled[43,44] and with unexpected, unnecessary antibiotics[41,45,46]. Patients' lack of participation in consultations and 'unvoiced agendas' were associated with misunderstandings, unnecessary and unwanted prescriptions and poor adherence[47].

The STAR program will provide clinicians with training to: 1) ask specific assessment questions in order to 'Lift the Lid' on patient's unvoiced agendas[48], and 2) use the 'Elicit-Provide-Elicit' strategy to identify and respond to expectations and information needs appropriately and in good time[49].

Patient behaviour change is addressed by consultation strategies that encourage clinicians to avoid a standard 'mini-lecture' about viruses, bacteria and antibiotics, in favor of eliciting key patient expectations and providing them with tailored information about the 'why' and 'how' of self care[34,35].

### Innovation in training clinicians

We developed and piloted two novel training methods that form the backbone of the STAR Program. They draw on the literature on clinician behaviour change and maximize the potential for acquiring and using new skills on a widespread scale. The 'context-bound learning method' is a bottom-up, adult learning, experiential approach that relies on clinicians themselves evaluating the importance of the issue and then reflecting on authentic case scenarios. Clinicians report that powerful learning flows from reflecting on their own consultations[34,50]. We have also developed a self-directed blended e-learning program that allows learners electronic access to video-rich clinical challenges before and after face-to-face training. This method has potentially wide applicability at low cost, and may assist in maintaining change.

### Study intervention

**Figure 1:**
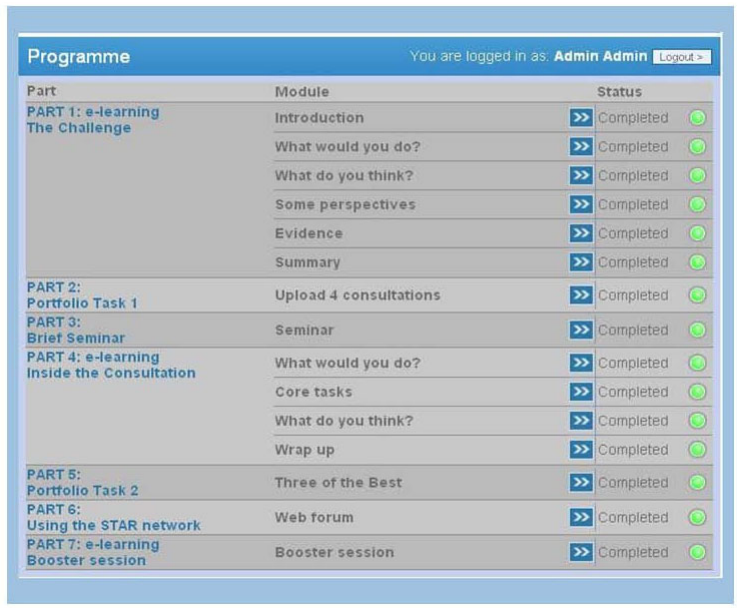
**The STAR Educational Program**.

The experimental intervention (the STAR Educational Program) will take clinicians through a course that will last approximately five hours. The course uses a wide range of replicable learning methods (didactic, interactive, reflective learning in groups and as individuals) focused on two themes: firstly, practice prescribing and resistance, and secondly, achieving consistency in messages to patients by learning new ways of handling everyday consultation challenges. Access to e-learning will be via the web. Clinicians will be given the choice of whether or not to share with other study participants their responses to the attitude questions, as well as their judgments on the different case scenarios contained within the software. Each clinician will work on an unfolding portfolio of learning that combines their e-learning judgments with other material. The program consists of seven parts (see Figure 1):

#### Part 1 (online)

Clinicians will be presented with four case scenarios and will be asked to make judgments about how they might handle these consultations. They will also respond to questions about their attitudes to resistance and antibiotic prescribing. They will have access to up-to-date summaries of research evidence, as well as videos giving a range of opinions (expert, patient and clinician viewpoint). The aim of this first part of the program is to heighten awareness and encourage the learner to consider their management of common infections, their own practice data on prescribing levels and resistance, practice circumstances and typical choice of antibiotic class.

#### Part 2 (online)

Clinicians will record and reflect on four cases involving infections and their decision whether or not to prescribe antibiotics. This will be included in their portfolio.

#### Part 3 (face-to- face)

Practice prescribers will come together for a seminar facilitated by a STAR study trainer. This seminar will have three components: reflections on the on-line learning, feedback and discussion on prescribing data and on antimicrobial resistance data from samples submitted by their practice over a five to ten year period (depending on availability of data). Clinicians will also be given the opportunity to reflect on the importance they attach to changing their antibiotic prescribing, as well as the challenges they face.

#### Part 4 (online)

Clinicians will be asked to repeat the four case scenarios, as well as the attitude questions from Part 1, and will be able to access other participating clinicians' responses. Key aspects of the principles of good communication in an antibiotic prescribing consultation will be reinforced. Clinicians will consider four video scenarios demonstrating key consulting strategies and they will be encouraged to examine the consultations for evidence of 'good practice in an antibiotic consultation'. After they have viewed each consultation they will be able to view video sequences where the patient, the clinician and an expert colleague give their perspectives on the consultation. There will also be links to supporting evidence.

#### Part 5 (online)

Clinicians will be asked to describe three of their consultations where they used the new strategies with patients consulting in usual care conditions. They will reflect on how they felt, what went well, and on additional novel ways for solving challenges. These reflections will be recorded on-line and can be securely shared with other clinicians on the program in a moderated web forum.

#### Part 6 (online)

An ongoing, active web forum will provide updates on emerging evidence and allow educators to respond to clinicians' queries, feedback and comments. Clinicians will be able to start new topics on the forum as well as respond to topics posted by clinical colleagues.

#### Part 7 (online)

A booster module will be available 6–8 months after completing the initial training, which will remind clinicians of the key strategies and the main messages of the study and encourage further reflection. This will consist of an online session reminding clinicians of the STAR consultation strategies and a video consultation for a common infection where clinicians are asked to identify key strategies used during the consultation. Clinicians will also be sent a snapshot of practice antibiotic prescribing rates from two recent winter months, compared with the corresponding months for the preceding year, before they were exposed to the intervention.

### Research objectives and outcome measures

#### Primary Objective

to assess whether exposing prescribers in general practices to the STAR Program results in fewer antibiotics being dispensed to the practice's patients. This will be assessed by examining the total number of dispensed oral antibiotics (with examination of trend by quarter) per 1000 registered patients, for the year subsequent to the practice being exposed to the STAR Program, using Prescribing Audit Reports and Prescribing Catalogues data (PARC). PARC data have been used widely in research, although they do not capture private prescriptions for antibiotics (rare in Wales). The database contains data for dose and the number/volume of antibiotics, but not the defined daily dose. Increasing use of delayed prescribing makes dispensed antibiotics a better outcome measure than prescribed antibiotics, since dispensed antibiotics is a better proxy for consumed antibiotics.

#### Secondary Objectives

to investigate other outcomes related to prescribing. The following secondary outcome measures will be examined:

1. Hospital admission rates for possible complications of common infections. This will be obtained from routinely collected data contained in the Patient Episode Database for Wales (PEDW) which records inpatient/day case care for all patients in NHS Wales hospitals and for Welsh residents treated elsewhere in the UK.

2. Microbiological sampling rates and proportion of resistant organisms isolated from urine. These data are held on the All-Wales Microbiology Database (DataStore).

3. Summary data will be collected for a sample of practices through extraction queries run on routinely collected data from GP computer systems. This will be used to examine:

▪ For patients consulting with selected upper and lower respiratory tract infections; a) complication rates b) rates of re-consultations for the same illness episode and for future similar illness episodes over the following 7 days, 14 days and 31 days c) the difference between prescribed and dispensed antibiotics.

4. We will compare the primary outcome (PARC dispensing data) for practices in the study, both control and intervention groups, with other Welsh practices outside the study.

5. A cost effectiveness analysis from an NHS perspective will be undertaken.

6. A process evaluation of study participants will be carried out in the intervention group.

## Method and design

The study is a randomized controlled trial with general practices as the unit of randomization and analysis.

### Sample size

The SD of year-to-year within-practice changes in total dispensed antibiotic rates per 1000 patients between 2001 and 2002 was 70 items per 1000 patients in Wales. To detect a difference of 10% (73 dispensed antibiotics per 1000 patients) in the change in total antibiotic dispensing rate per annum between intervention and control practices, with 90% power, requires 21 practices per group. However, increasing this to 30 practices per group (approximately 80–90 clinicians per group) will allow a more robust exploration of a) practice characteristics and prescribing outcomes and b) the effect on resistance in urine samples: 25 practices per group will give 90% power to detect a reduction in resistance rates from the current 25% to 20% for Trimethoprim and from the current 6% to 3% for Cefalexin.

### Recruitment

A total of 60 practices will be recruited. Assuming an average practice size of 5 GPs, we estimate around 160–200 GPs and Nurse Practitioners will take part. The aim is to recruit all these practices in the first 6 months of the study. Due to the nature of the intervention, practices must be adequately computerized and participants will have to work at least 5 clinical sessions per week to take part in the STAR Educational Program. We will attempt to recruit at least two thirds of the prescribers at each practice. This is due to the nature of the analysis and the feedback provided on an individual and practice level. Practices who submit samples to laboratories that are not part of the DataStore system will be excluded from the study. GPs and nurse practitioners in Wales will receive an invitation letter, information sheet, personalized consent form, return envelope, and a flyer on the study. Additionally, letters to practice managers will be sent to alert them to the study.

### Randomization

Randomization will take place once all practices have been recruited in order to obtain an allocation with near optimal balance for total annual antibiotic prescribing averaged over the past three years (PARC), practice size (number of whole time equivalent staff at recruitment) and proportion of GPs in the practice registered for the study. Practices will be sub-divided into three blocks and allocation balanced within each block[51,52]. All possible allocations within a set will be estimated and the balance statistic based on the sum of the squares of the differences between each group on each measure (standardized). The 1000 possible allocations within each set with the best balance will be selected and passed to an independent statistician and one allocation will be selected at random. Once the final allocation to two groups has been selected for each set of practices, this independent statistician will randomly allocate intervention or control to the two groups.

### Trial procedures

The STAR Program will be delivered to intervention practices within the first study year, and then offered to control practices after the follow-up is complete. Practices will be followed up one year after the intervention.

### Process evaluation

We will conduct a process evaluation in which we:

1. Explore participation in the seminars and use of software supported learning and map the clinicians' use of the system (how often they log in, which pages they use) in relation to the primary outcome.

2. Complete qualitative interviews with one clinician from each participating intervention practice to explore ongoing use of the prescribing and resistance data, the website, the communication skills, and the acceptability and perceived usefulness of various components of the intervention. Clinicians from intervention practices will be purposively sampled for interview based on gender, experience, antibiotic prescribing, practice setting and when they completed the training. Interviews will continue until all the themes are saturated, previous experience suggests that this will be around 30 interviews[35,38,53]. The interviews will be conducted over the telephone and audio recorded. The interviews will be transcribed for later analysis.

### Health economics evaluation

We will also undertake a cost effectiveness analysis from an NHS perspective. Direct costs/savings will include the cost of the STAR Program, potentially longer consultations in intervention practices and potentially fewer antibiotics dispensed. Indirect costs/savings will include differences in antibiotic adverse events, re-consultations for the same illness and complications of common infections including hospital admissions. All resource use will be monitored prospectively. Since this is a 'complex intervention', no attempt will be made to examine the cost effectiveness of each specific component. Prospective monitoring of health service resource use, however, will identify the cost of each element of the intervention. The different elements will also be examined in the qualitative study. Education represents a one-off cost which may produce a stream of benefits over time. Accordingly, the costs of education can be dealt with similarly to capital equipment with costs expressed on an Equivalent Annual Cost (EAC) basis[54]. Length of consultation will be estimated using facilities in existing practice computer systems in a sample of practices.

The study has been approved by the Multi-centre Research Ethics Committee (MREC 06/MRE09/31) and all Local Health Boards (LHBs) in Wales.

### Analysis

#### Main analysis

The main analysis will be intention to treat and will compare the two groups for annual total antibiotics dispensed per 1000 practice patients within practices in the year following the intervention, using analysis of covariance with the average of the previous three years' prescribing as a covariate. Secondary outcomes of average hospital admission rates for specified complications, antibiotic resistance rates will be similarly compared between the two groups for the whole year. A comparison will be made between groups for complications managed in primary care, re-consultations and further consultations for a similar illness, using summary data extracted from practice computer systems, for a subset of practices. Exploratory analyses will use analysis of covariance to adjust for practice factors that might plausibly influence outcomes. Weighted average Townsend scores for each practice will be used to examine socio-economic influences on prescribing. The degree of uptake of the intervention will be used in further exploratory models to explore impact on outcomes. Antibiotics dispensed for intervention and control practices will be compared to Welsh practices not participating in the study (those not consented and those not approached) to assess non-specific effects of taking part in a trial as well as the external validity of the trial.

#### Qualitative analysis

We will employ standard thematic analysis techniques, where transcripts will be closely examined to identify themes and categories[55]. Codes will be applied to these broad themes which will then be broken down further into sub-codes. Agreement on concepts and coding will be sought between members of the research team in order to ensure reliability. A proportion of the data (10%) will be coded by two different team members to check on reliability of the coding scheme. The interviewing will be iterative, where new themes emerge we will incorporate them into the interviews. Thematic analysis will be supported by the use of computer-assisted qualitative analysis software (NVIVO8).

#### Cost effectiveness analysis

All resources will be valued using conventional methods[54]. Totals, means and confidence intervals for each training component will be identified, and analyzed using SPSS. Where costs are skewed, bootstrapped confidence intervals will be used.

The net cost of the intervention (likely to be positive) will be assessed against the primary outcome (reduction in dispensed oral antibiotics) to provide an incremental cost effectiveness ratio (ICER). Although there is a literature on the effectiveness of measures to improve antibiotic use, we could identify no comprehensive cost effectiveness analyses against which the above ICER can be compared. Accordingly the present study will not be able to provide evidence to show whether STAR is more or less cost effective than other measures with the same objective. It will, however, provide an evidence base for comparison in future studies.

## Discussion

This trial will be the first to evaluate the effectiveness of a theory-based intervention involving a blended learning program which focuses on both clinicians' reflections on their prescribing habits and practice level data on prescribing and antimicrobial resistance, as well as an introduction to novel consulting strategies for use during consultations for common infections. This approach has the potential to improve communication between clinicians and patients, as well as reducing unnecessary antibiotic prescribing. One recent study which focused on improving communication achieved an impressive 40% relative reduction in antibiotic prescribing at 12 months[56].

### Strengths

This study will be implemented in Wales and will include general practice surgeries from areas of high social deprivation as well as more affluent areas. We will use routine data as the main outcome which will enable us to compare practices taking part in the study with those outside the study. We will also be able to look at dispensing data both before and after the study to examine changes in behaviour.

Given that in the UK in recent years there has been a considerable reduction in inappropriate antibiotic prescribing, a reduction of 10% in antibiotic prescribing rates may seem optimistic. However, taking into account the findings of Welschen[16,33] and Altiner[56] and the fact that the UK has higher rates of antibiotic prescribing than both the Netherlands and Germany, and that the intervention we propose uses an innovative mix of approaches to behaviour change, we feel that this is nevertheless feasible.

The main outcome will not simply be antibiotic prescribing in a single consultation as in individually randomized trials[57], but rather, antibiotic prescribing for a whole year. This will allow for assessment of maintenance of skills and more powerful analyses that better assess the pragmatic effect of the intervention over time.

The cluster design will limit the chances of contamination. Once individual practitioners are trained in new consulting skills and are given new information about the indication and choice of antibiotic, they could not effectively revert back to their previous 'usual care' according to individual patient randomization. All participating clinicians in the same practice will be randomized to the same intervention condition to prevent contamination within practices.

### Protecting against bias

Practices who volunteer for the study are likely to be motivated to have access to the intervention, so those who are allocated to the control group may be disappointed. In order to avoid differential dropout between the experimental and control groups, we will offer those practices in the control group the opportunity to complete the training program after the one year follow-up period.

One important issue is whether clinicians will actually use the skills that they have learned in practice, since maintenance of these skills is challenging. Whilst the clinicians will learn the theory behind the communication skills and will learn to use the key strategies during consultations and appreciate the reasons for doing things this way, they might still not incorporate these into their routine practice. However, the intervention focuses on why change is important while at the same time enhancing clinicians' skills. The strategies taught will fit within the timescale of a routine consultation and this we hope will increase uptake. In addition, we will have a 'booster', which will consist of both on and offline elements to encourage maintenance of skills.

### Evaluations

The study will incorporate both an economic evaluation and a process evaluation. The process evaluation will allow us to explore the impact of different aspects of the intervention. If the trial does not show an effect it will allow us to explore possible reasons for this. In addition to estimating the overall cost of the intervention, the economic evaluation will identify its effect on consultation length as this is likely to be a factor of concern to clinicians which could affect its adoption. One challenge we face is measuring the impact of using the communication skills on consultation length. We plan to use the practice computer systems to provide an estimate of this, where possible.

The STAR Program grew out of seminal qualitative studies and intervention development research on clinician behaviour change. This study will therefore not simply make an incremental contribution. It will evaluate a unique, yet applicable, approach that has the potential to radically change research direction, clinical practice and policy. Since enhanced communication skills are the core of the intervention, beneficial effects on clinical practice are likely, not only in the management of common infections, but also in other clinical areas. The intervention is easily generalisable and could be rolled out using current technology and prescribing advisors to deliver the seminars. The intervention design using novel blended learning approaches will be applicable to continuing professional development in other clinical domains.

## Competing interests

The authors declare that they have no competing interests.

## Authors' contributions

SS led the writing of this manuscript and developed the blended learning program, together with Stephen Rollnick. She also contributed to the design of the study. CB is the Principal Investigator, conceived the study and led the study design and funding application. KH is a trial statistician and contributed to the study design, and analysis plan. FD is a trial statistician and contributed to study design, power calculation and analysis plan. DC is a health economist and contributed to study design and the health economics component. ME is a clinical epidemiologist, he contributed to design of the study. SR is a clinical psychologist who contributed to study design and the development of the intervention. LM contributed to the design of the study. MJB and MH are the trial managers and contributed to the manuscript. JE is the data manager who contributed to the manuscript. All authors contributed to, read and approved the final version of the manuscript.

## Pre-publication history

The pre-publication history for this paper can be accessed here:


